# Anaphylaxis- don't forget lupin!

**DOI:** 10.1186/2045-7022-3-S3-P158

**Published:** 2013-07-25

**Authors:** M Makatsori, A Cappella, R McKenzie, I Skypala

**Affiliations:** 1 Allergy Department, Royal Brompton Hospital, London, UK

## Background

Lupin is a decorative flower found in gardens around the world. It is a member of the legume family and in recent years, flour produced from its seeds has been increasingly used in food manufacturing, especially in breads, baked products and confectionery. Within a similar timeframe, lupin allergy has emerged as a major problem in European countries, with more cases also now appearing in the United Kingdom.

## Methods

A 46 year old female with a history of asthma and seasonal allergic rhinitis was referred to our Food Allergy clinic. In the previous 2 years, she suffered two episodes of anaphylaxis without being able to identify the cause. The first anaphylactic episode occurred after eating a crab canapé. The other, after eating duck pâté, beef with vegetables and a fruit tart. After eating this meal, she developed a sensation of throat tightness, nausea and felt unwell. Twenty minutes later, she went on to develop wheeze and stridor, generalised pruritus, erythema, vomiting and diarrhoea. She has had milder symptoms after eating lobster chowder and mussels in Belgium and also with chocolate and toffee. She has been avoiding shellfish since the reaction. She was also avoiding peanuts as she was found to have a positive specific IgE test in the general allergy clinic.

## Results

Total IgE was 159iu/ml. Specific IgE test to lobster, mussel, shrimp, fish, lipid-transfer protein, tree nuts and various legumes were negative. Specific IgE to peanut was 1.02iu/ml. Ara h 1, 3, 8 and 9 were negative while ara h2 was 1.74 iu/ml. Skin prick testing (SPT) to peanut reagent, raw peanut and roasted peanut were negative. SPT to lupin flour was 25mm and specific IgE 42iu/ml. Soy flour was 4mm. She has been eating soy and tolerates other legumes. SPT to tragacanth gum, a legume that she was using in sugar flower making was negative.

## Conclusion

The strongly positive SPT and specific IgE tests to lupin, in conjunction with this being the common ingredient across the foods our patient reacted to, suggest lupin as the cause of these reactions. It is important for clinicians to consider lupin as a potential cause of unexplained food-related allergic reactions, as its use has now become widespread throughout Europe. Furthermore, cross-reactivity to peanut should be clarified with challenge testing. Of note, our patient had been using a facial anti-wrinkle cream that contained white lupin seed extract for over 7 years. This may represent an additional route of sensitisation.

## Disclosure of interest

None declared.

